# Prediction of *n*-octanol/water partition coefficients and acidity constants (*pK*_*a*_) in the SAMPL7 blind challenge with the IEFPCM-MST model

**DOI:** 10.1007/s10822-021-00394-6

**Published:** 2021-07-10

**Authors:** Antonio Viayna, Silvana Pinheiro, Carles Curutchet, F. Javier Luque, William J. Zamora

**Affiliations:** 1 Department of Nutrition, Food Sciences and Gastronomy, Faculty of Pharmacy and Food Sciences, Institute of Biomedicine (IBUB), and Institute of Theoretical and Computational Chemistry (IQTC-UB), University of Barcelona (UB), Avda. Prat de La Riba, 171, 08921 Santa Coloma de Gramenet, Spain; 2 Institute of Exact and Natural Sciences, Federal University of Pará, Belém, Pará, 66075-110 Brazil; 3 Department of Pharmacy and Pharmaceutical Technology and Physical Chemistry, Faculty of Pharmacy and Food Sciences, and Institute of Theoretical and Computational Chemistry (IQTC-UB), University of Barcelona, Av. de Joan XXIII, 27-31, 08028 Barcelona, Spain; 4 School of Chemistry and Faculty of Pharmacy, University of Costa Rica, San Pedro, San José Costa Rica; 5 Advanced Computing Lab (CNCA), National High Technology Center (CeNAT), Pavas, San José Costa Rica

**Keywords:** SAMPL7, Physical properties, Water-octanol log *P*, p*K*_a_, Solvation free energy, MST model, Continuum solvation models, Conformational study

## Abstract

Within the scope of SAMPL7 challenge for predicting physical properties, the Integral Equation Formalism of the Miertus-Scrocco-Tomasi (IEFPCM/MST) continuum solvation model has been used for the blind prediction of *n*-octanol/water partition coefficients and acidity constants of a set of 22 and 20 sulfonamide-containing compounds, respectively. The log *P* and p*K*_a_ were computed using the B3LPYP/6-31G(d) parametrized version of the IEFPCM/MST model. The performance of our method for partition coefficients yielded a root-mean square error of 1.03 (log *P* units), placing this method among the most accurate theoretical approaches in the comparison with both globally (rank 8th) and physical (rank 2nd) methods. On the other hand, the deviation between predicted and experimental p*K*_a_ values was 1.32 log units, obtaining the second best-ranked submission. Though this highlights the reliability of the IEFPCM/MST model for predicting the partitioning and the acid dissociation constant of drug-like compounds compound, the results are discussed to identify potential weaknesses and improve the performance of the method.

**Supplementary Information:**

The online version contains supplementary material available at 10.1007/s10822-021-00394-6.

## Introduction

Lipophilicity and (de)protonation are physicochemical properties that play a fundamental role to understand the biological activity of drugs [1–4]. From a pharmacokinetic point of view, these properties exert a marked influence on the ADME-Tox profile of drugs, affecting solubility in physiological fluids and permeability through biological barriers, as well as the excretion rate from the human body [5]. With regard to drug pharmacodynamics, lipophilicity affects recognition and binding of drugs to their macromolecular targets, since the global hydrophobic character is related to the changes in (de)solvation involved in ligand binding, whereas a complementarity between the 3D distribution of hydrophobic/hydrophilic regions in the drug and the binding pocket should reinforce the drug-target interaction [6–8]. On the other hand, the (de)protonation of a compound can clearly exert influence on the bioavailability of a molecule, affecting not only the biodistribution of the bioactive compound in the organism, but altering the interaction pattern that may be formed with specific residues in the binding pocket [9, 10].

**Scheme 1:**
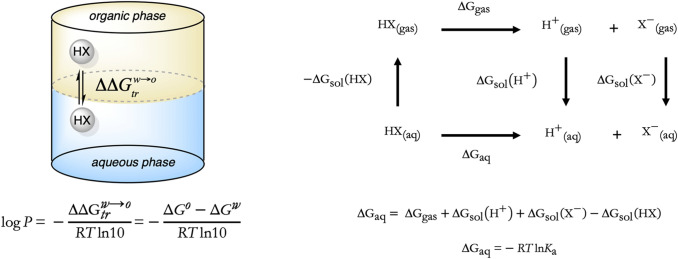
Thermodynamic cycles used to determine (left) the transfer free energy of a neutral (HX) compound between *n*-octanol and water, and (right) the p*K*_a_ estimation of a titratable compound, where HX and X^−^ stand for the acidic and basic species, respectively

The *n*-octanol/water partition coefficient (log *P*) is the physicochemical parameter generally adopted to quantify the lipophilicity of a compound, and can be experimentally determined from the partitioning between aqueous and *n*-octanol phases. From a computational point of view, log *P* can be estimated from the transfer free energy ($\Delta \Delta {\text{G}}^{{{\text{w}} \to {\text{o}}}}$; Scheme 1) of the molecule between these two solvents, which in turn can be derived from the solvation free energy in *n*-octanol ($\Delta{\text{G}}_{\text{solv}}^{\text{o}}$) and water ($\Delta{\text{G}}_{\text{hyd}}^{\text{w}}$). The ionization equilibrium of a titratable compound is quantified by the negative logarithm of the acid dissociation constant (p*K*_a_), which reflects the population of acidic and basic species. This quantity can be related to the free energy change for the ionization of the compound in water ($\Delta{\text{G}}_{\text{aq}}$; Scheme 1), which in turn can be calculated combining the free energy change for this process in the gas phase with the solvation free energies of protonated (HX) and deprotonated (X^−^) species of the compound and the solvation free energy of the proton [11, 12].

The availability of computational tools able to provide accurate estimates of log *P* and p*K*_a_ is valuable to provide useful guides in the search of novel *hit* compounds and the drug development process [13, 14]. This may deserve special interest in the screening of large libraries of compounds, as the experimental measurement of these properties would be demanding and often facing experimental challenges for specific classes of compounds. In this context, we present here the results obtained in the context of the SAMPL7 blind challenge [15]. Given the fundamental role of the solvation free energy in the computational prediction of both log *P* and p*K*_a_, our computational strategy exploits the B3LYP/6-31G(d) parametrized version [16, 17] of the quantum mechanical IEFPCM/MST solvation model [18], which relies on the Integral Equation Formalism of the Polarizable Continuum model [19, 20]. Here, we report the results obtained for predicting the log *P* and p*K*_a_ for a group of sulfonamide-containing compounds. The results are discussed in light of the experimental data provided by the organizers of SAMPL7 [21] and the theoretical estimates reported by others groups, as well as with the IEFPCM/MST results obtained in previous editions of this contest [22, 23].

## Methods

### Test compounds

**Fig. 1:**
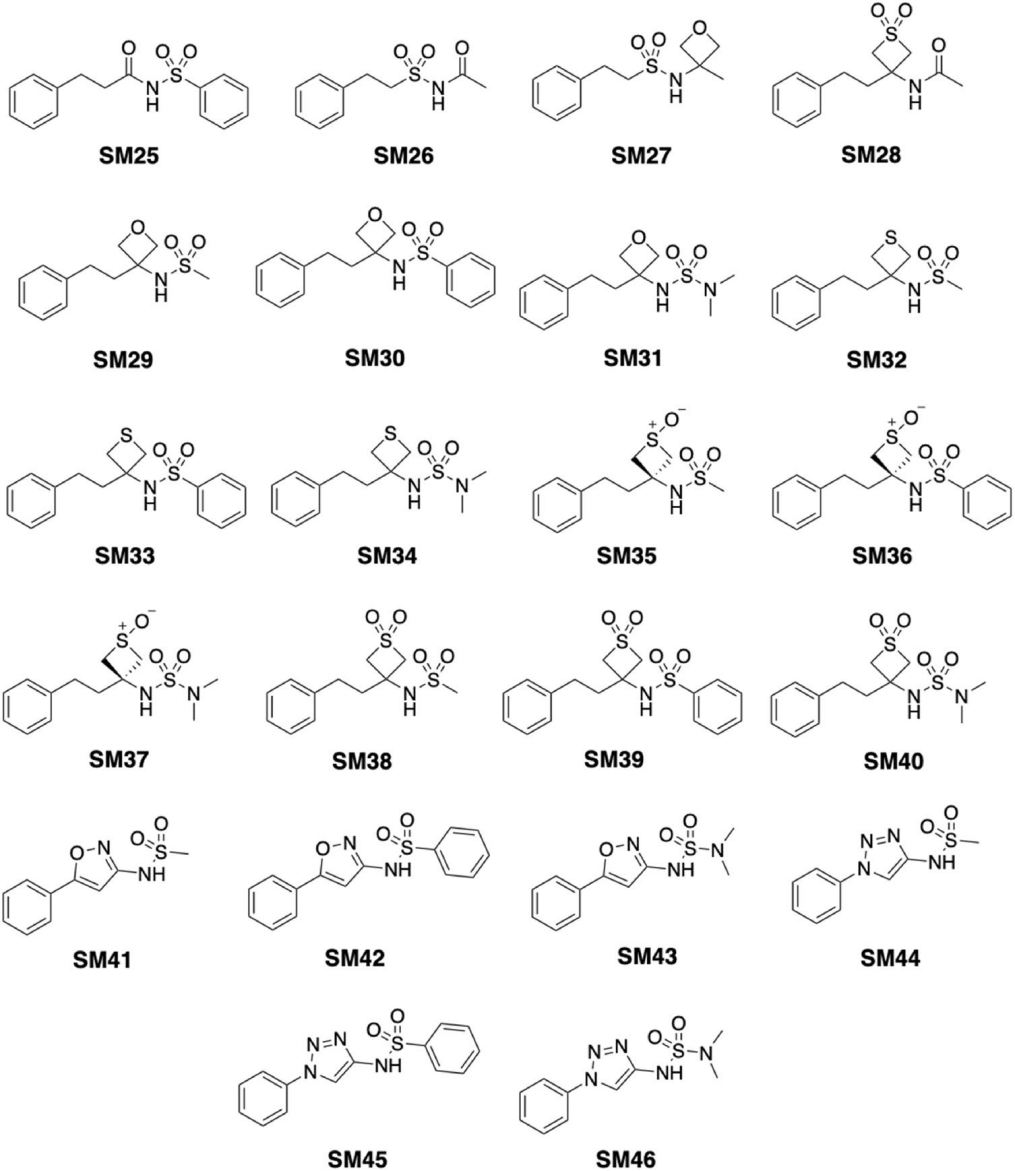
Dataset of 22 small molecules proposed in the SAMPL7 log *P* challenge

The dataset used in the SAMPL7 challenge contains 22 compounds (numbered SM25 to SM46; Fig. 1) provided by Carlo Ballatore and coworkers at UCSD (University of California, San Diego). Most of the compounds share chemical motifs, including the presence of a sulfonamide unit, a phenylethyl moiety (with the exception of compounds SM41- SM46), and a four-membered ring fused to the main chain, often containing oxygen and sulphur. Few compounds (SM41-SM46) include specific moieties, such as isoxazole (SM41-SM43) and triazole (SM44-SM46), in the main chain. Finally, besides the sulfonamide group, certain compounds contain sulfoxide (SM35-SM37) or sulfone (SM38-SM40) groups in their chemical structure. The *smiles* codes of the 22 compounds were obtained from the SAMPL7 website [15], and used to generate their 3D geometries with OpenBabel [24].

### Log P computation

A preliminary sampling of the conformational preferences of the compounds was performed with Frog 2.14 [25]. Let us note that this program not only generates conformations at a reduced computational cost, but also exhibits a high performance in generating conformations close to the bioactive species, as noted in a rmsd 0.74 ± 0.44 Å for 85 drug-like compounds (Astex dataset), and a median rmsd below 1 Å for a subset of compounds containing up to 7 rotatable bonds [25]. On the basis of the structural complexity of the molecules, generation of conformations was limited to a maximum of 20 conformers, which were visually checked in order to eliminate redundant conformations. The geometry of the conformers in water and *n*-octanol was optimized at the B3LYP/6-31G(d) level of theory [26, 27] taking into account solvent effects on the geometrical parameters with the IEFPCM/MST model, which was implemented in a local version of Gaussian 16 [28]. The minimum energy nature of the optimized geometries in each solvent was verified upon inspection of the vibrational frequencies, and conformations displaying negative frequencies were discarded. Thermal corrections determined in water and *n*-octanol were subsequently added to estimate the relative free energy of conformations in the two solvents. Finally, single-point energy calculations in the gas phase were performed to estimate the solvation free energy of each conformation. Then, the log *P* was determined considering the Boltzmann-weighted population of the conformational families obtained in water and *n*-octanol.

### pK_a_ computation

The p*K*_*a*_ of the deprotonation equilibria between acid and basic microstates was based on the thermodynamic cycle shown in Scheme 1. The ensemble of conformations determined in water for the set of compounds was used as starting geometries to build up the species involved in the deprotonation equilibria, according to the information provided by the SAMPL7 organizers for the different microstates [15]. The addition/removal of hydrogen atoms from the starting geometry of conformers was done manually using GaussView 6 (i.e., the graphical interface of Gaussian software) [29]. The geometries were optimized at the B3LYP/6-31G(d) level of theory taking into account hydration effects with the IEFPCM/MST model. The free energy difference between protonated and deprotonated species was estimated by combining the relative energies determined with single-point computations performed at the MP2/aug-cc-pVDZ level of theory [30] with solvation free energies and thermal corrections to the free energy calculated at the B3LYP/6-31G(d) in water. The p*K*_a_ was determined using the experimental free energy of the proton in water (− 270.29 kcal/mol), which was determined by combining the gas phase free energy (− 6.28 kcal/mol), the free energy correction from 1 atm and 298 K to 1 M and 298 K state (1.89 kcal/mol), and the hydration free energy of the proton (− 265.9 kcal/mol) [31]. Finally, a Boltzmann weighting scheme was applied to account for the relative stabilities of the conformational species determined for the microstates involved in the deprotonation reaction, following the computational strategy adopted in previous studies [32, 33].

### Raw data

The datasets generated during and/or analysed during the current study are available in the SAMPL7-IEF-PCM-MST GitHub repository [34].

## Results and discussion

### Log P prediction

The predicted log *P* values are listed in Table 1. The root-mean square deviation (rmsd) between IEFPCM/MST results and experimental data is 1.03 log units, which places our results among the most accurate values in the comparison with both physical (rank 2nd) and global (comprising all submissions within empirical and physical categories; rank 8th) methods [21], taking into account the small differences observed between methods with rmsd ≤ 1 (see Supporting Information Fig. S1). The best ranked QM-based solvation models (see Supporting Information Fig. S2) were the *Cosmotherm* version of COSMO-RS [35] (ID *COSMO RS*, rmsd = 0.78), our method (ID *TFE IEFPCM MST*, rmsd = 1.03), the NHLBI TZVP model (ID *TFE NHLBI TZVP QM*, rmsd = 1.55), which combined B3LYP/Def2-TZVP computations in the gas phase with solvent effects determined using the SMD solvation model [36], the 3D integral equation theory with a cluster embedding approach [37] (ID *EC RISM wet*, rmsd = 1.84), and another model that combined B3LYP computations with dispersion corrections in the gas phase with the SMD model [36] (ID *TFE b3lyp3d*, rmsd = 2.19), reflecting a performance similar to the trends found in the SAMPL6 challenge [38].

**Table 1:** Calculated (ID *TFE IEFPCM MST*) and experimental *n*-octanol/water partition coefficient (log *P*) determined for the set of compounds included in the SAMPL7 dataset

Compound	Calculated	Experimental^a^	Δlog *P* (calc − exptl)
SM25	1.89	2.67	− 0.78
SM26	− 0.21	1.04	− 1.25
SM27	1.76	1.56	0.20
SM28	0.83	1.18	− 0.35
SM29	1.24	1.61	− 0.37
SM30	3.54	2.76	0.78
SM31	1.62	1.96	− 0.34
SM32	1.64	2.44	− 0.80
SM33	4.29	2.96	1.33
SM34	2.40	2.83	− 0.43
SM35	0.77	0.88	− 0.11
SM36	3.75	0.76	**2.99**
SM37	1.88	1.45	0.43
SM38	0.48	1.03	− 0.55
SM39	2.48	1.89	0.59
SM40	1.43	1.83	− 0.40
SM41	0.88	0.58	0.30
SM42	3.75	1.76	**1.99**
SM43	1.85	0.85	1.00
SM44	− 0.16	1.16	− 1.32
SM45	2.04	2.55	− 0.51
SM46	0.95	1.72	− 0.77
mse^b^	− 0.07		
mue^b^	0.80		
rmsd^b^	1.03		

Bold values indicate compounds with the largest deviation (> 1.50 log *P* units) between predicted and experimental values

^a^See [39]

^b^Mean signed error (mse), mean unsigned error (mue), and root-mean square deviation (rmsd) calculated relative to the experimental values (log *P* units)

**Fig. 2:**
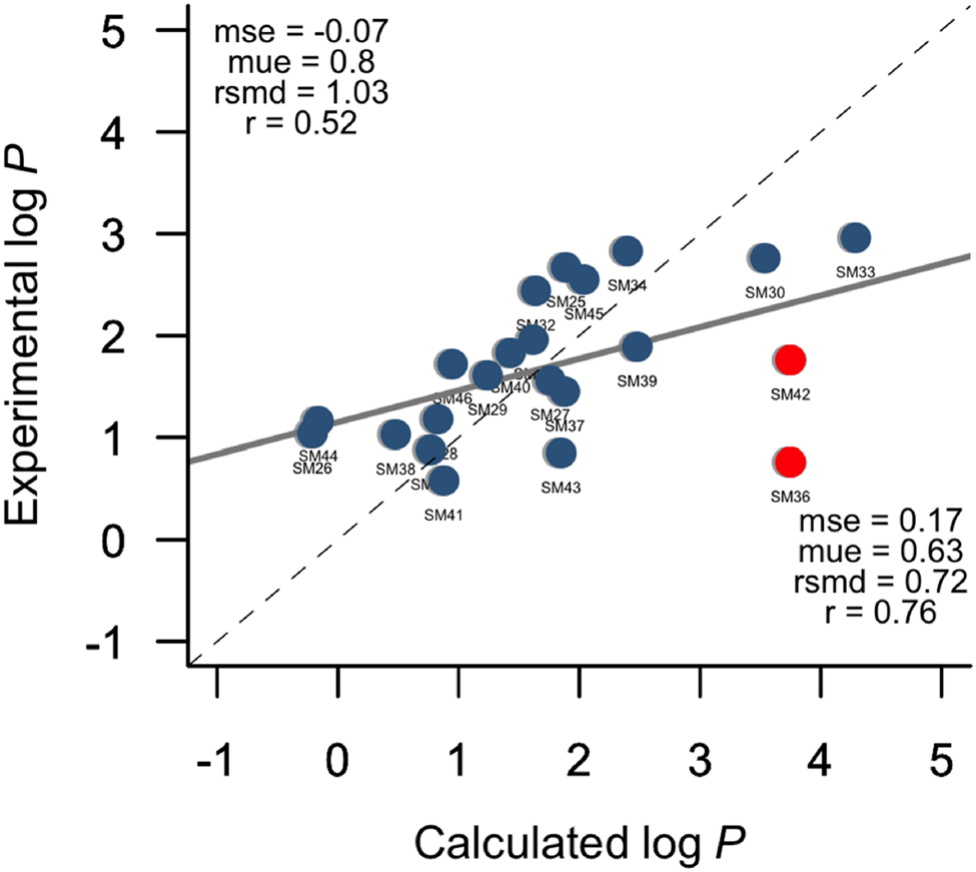
Comparison between experimental and IEFPCM/MST *n*-octanol/water log *P* for the SAMPL7 dataset. Red points represent the compounds with the largest errors in the original submission. Statistical analyses are shown for (top left) all compounds and (bottom right) after exclusion of SM36 and SM42

The largest deviations (> 1.50 log *P* units) between predicted and experimental log *P* values are found for SM36 and SM42 (see Table 1). These deviations are in line with the analysis of the compounds that presented the highest mean absolute error between computed and experimental values (see Supporting Information Fig. S3), since SM42 and SM36 are in ranks 1 and 5, respectively. Upon exclusion of these compounds, the rmsd is reduced to 0.72 log *P* units, and the correlation between calculated and experimental values improves from 0.52 to 0.76 (see Fig. 2).

Compared to SM35 and SM41, SM36 and SM42 imply the replacement of a methyl group by a phenyl substituent, which would increase the hydrophobicity of the compound. This trend is reflected in the experimental log *P* values for pairs SM41-SM42, SM29-SM30, SM32-SM33, SM38-SM39 and SM44-SM45, where the methyl-phenyl replacement leads to an average increase of 1.02 log *P* units. In this context, the pair SM35-SM36 shows a distinctive trait, as the log *P* is decreased by − 0.12. In fact, more than 80% of submissions predicted the log *P* of SM36 and SM42 to be larger compared to the log *P* of SM35 and SM41, respectively (see Supporting Information Fig. S4).

**Fig. 3:**
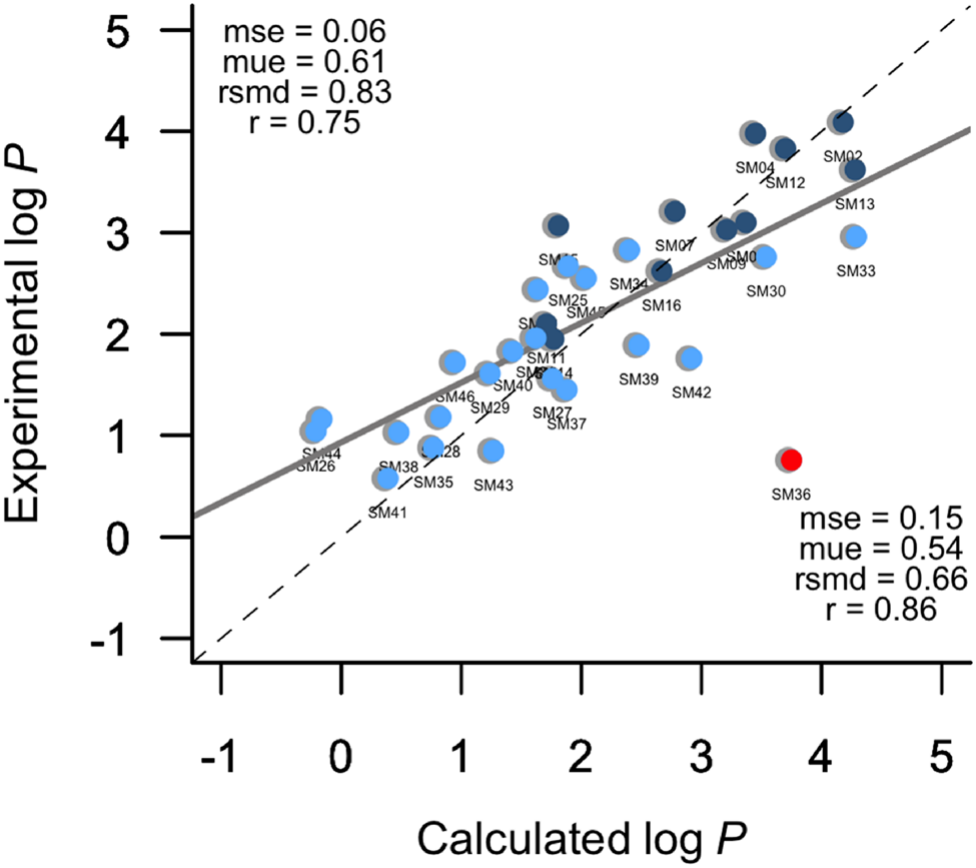
Comparison between experimental and IEFPCM/MST *n*-octanol/water log *P* for the combined dataset including the 11 fragment-like small molecules in the SAMPL6 log *P* challenge (blue) and 22N-acylsulfonamides in the SAMPL7 log *P* challenge (lightblue). The red point represents the compound with the largest error in the final dataset. Statistical analyses are shown for (top left) all compounds and (bottom right) after exclusion of SM36

Finally, we have compared the predictions performed for the SAMPL7 dataset with the results obtained in the SAMPL6 edition, which comprised a series of 11 fragment-like small molecules [38]. Upon exclusion of SM36, the comparison yields an overall rmsd of 0.66 log *P* units (see Fig. 3). Therefore, assuming that the reported accuracy for log *P* determination is ~ 1 log unit, present results lend support to the reliability of the IEF-PCM/MST model and encourage future efforts for achieving a better description of solvation effects.

Without detracting from our values, among the set of methods presented in the current edition of log *P* SAMPL7 challenge, one may notice that methods based on Machine Learning (ML) have led to a better match with the experimental values provided by the organization. In our view, these type techniques present great advantages, since they allow a very quick estimation due to their low computational cost, making them suitable for large compound screening campaigns. However, the reliability of these methods may be affected by the chemical coverage of the data used in their training. In this context, QM-based methods seem better suited to provide a detailed analysis of the structural and energetic features of compounds, though this requires a significantly larger computational cost, which may be necessary in the analysis of compounds containing novel chemical scaffolds. Keeping in mind the vast diversity of the chemical space [40], it may be expected that integration of QM and ML techniques will be very powerful to enhance the quality and reliability of ML models in the prediction of physicochemical properties, enabling large-scale exploration of the chemical space [41, 42].

### pK_a_ prediction

Only physical methods contributed to predicting the p*K*_*a*_ values for the 22 sulfonamide-containing compounds included in the blind test. Table 2 reports the p*K*_a_ values estimated from IEFPCM/MST computations and submitted to SAMPL7. Compared to the values available with the SAMPL7 repository [39], the difference between the originally submitted results and those estimated by the organizers from the microstates reported in our original submission is in general within 0.10 p*K*_a_ units, except for SM37, where the difference increases up to 3.90 p*K*_a_ units (detailed values are available in Supporting Information Table S1). The origin of this difference was due to a mistake in the relative free energy reported by us for the negatively charged microstate of compound SM37, as we had flipped the values for microstates SM37_micro004 and SM37_micro005 in the file submitted to the SAMPL7 website. This mistake led to a different macroscopic p*K*_*a*_ value between the one calculated automatically by the organizers and the one reported in the original submission. For these reasons, we have kept the macroscopic p*K*_a_ value of the original submission in Table 2.

**Table 2:** Calculated (ID *IEFPCM MST*) and experimental p*K*_a_ determined for the set of compounds included in the SAMPL7 dataset

Compound	Calculated	Experimental^a^	Δp*K*_a_ (calc − exptl)
SM25	7.24/3.30	4.49	**2.75/**1.19
SM26	4.52	4.91	− 0.39
SM27	12.34	10.45	**1.89**
SM28	16.12	> 12.00	–
SM29	11.51	10.05	1.46
SM30	11.00	10.29	0.71
SM31	10.84	11.02	− 0.18
SM32	11.95	10.45	1.50
SM33	10.69	> 12.00	–
SM34	10.64	11.93	− 1.24
SM35	10.28	9.87	0.41
SM36	9.20	9.8	− 0.6
SM37	8.11	10.33	− **2.22**
SM38	9.82	9.44	0.38
SM39	8.85	10.22	− 1.37
SM40	8.26	9.58	− 1.32
SM41	5.13	5.22	− 0.09
SM42	4.86	6.62	− **1.76**
SM43	4.43	5.62	− 1.19
SM44	7.09	6.34	0.75
SM45	7.37	5.93	1.44
SM46	5.56	6.42	− 0.86
mse	0.00		
mue	1.13		
rmsd	1.32		

Bold values indicate the compounds with the largest deviation (> 1.50 in p*K*_a_ units) between theoretical and experimental values. For SM25, the value of the original submission and the corrected one during the revision of the calculated data are indicated as plain text and in italics, respectively

^a^Ref [43]

**Fig. 4:**
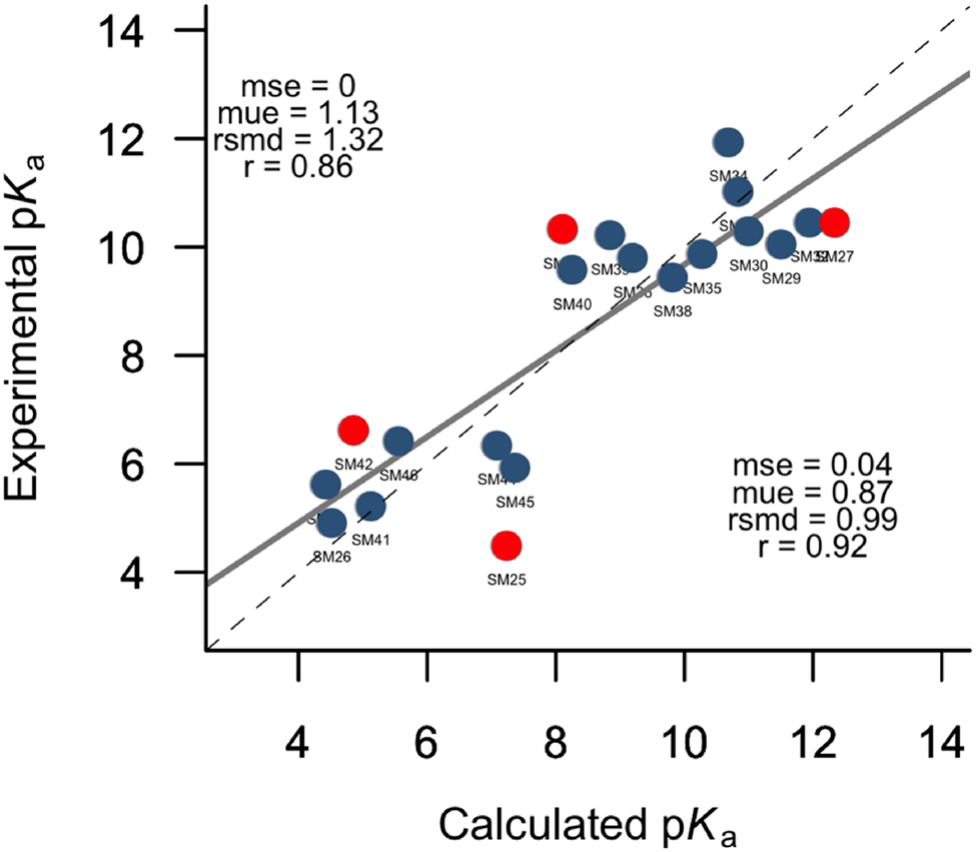
Comparison between experimental and IEFPCM/MST p*K*_a_ for the SAMPL7 Dataset. Red points denote compounds with the largest errors in the original submission. Statistical analyses are shown for (top left) all compounds and (bottom right) after exclusion of SM25, SM27, SM37 and SM42

The rmsd between predicted and experimental p*K*_*a*_ values is 1.32 log units, which places our results among the best-ranked submissions (rank 2nd, Supporting Information Fig. S5). The largest deviations (> 1.50 in p*K*_a_ units) involve four compounds: SM25, SM27, SM37 and SM42. Exclusion of these compounds reduces the rmsd to 0.98 p*K*_a_ units, and the correlation between calculated and experimental values changes from 0.86 to 0.92 (see Fig. 4).

**Fig. 5:**
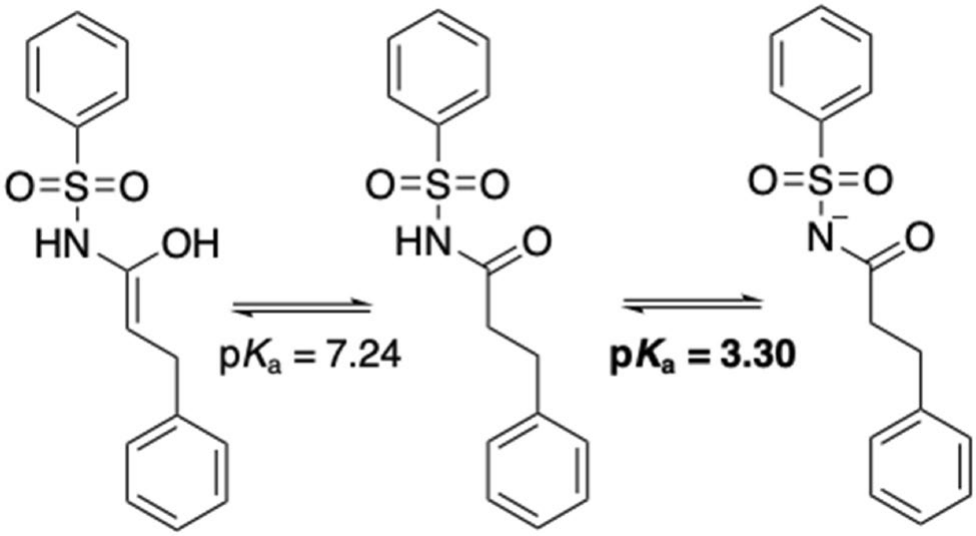
Microstates involved in the error of SM25 p*K*_*a*_ estimate

To explore the potential sources of these deviations, we compared the results obtained for SM25, SM27, SM37 and SM42 with the values reported by the contributors ranked 1st (ID *EC_RISM*) and 3rd (ID *TVZP_QM*) in the blind test (see Table 3). The results show that EC_RISM provides a range of values (5.42–10.17) that compares well with the experimental data (4.49–10.45), whereas our results are distributed in a slightly larger range (4.86 to 12.34). In contrast, the TVZP_QM values are in a narrower range (6.77–7.65). We then checked the workflow used to compute the macroscopic p*K*_*a*_ and found a mistake in the definition of the Boltzmann weights for the conformations sampled for the main microstates of compound SM25 (Fig. 5), which caused a 3.94 units decrease in the p*K*_a_ value (p*K*_a_ = 3.30), remaining at 1.19 units from the experimental value.

**Table 3:** Comparative results of the four highly deviated compounds with the first (ID *EC_RISM*) and third (ID *TZVP_QM*) ranked methods in the SAMPL7 p*K*_a_ challenge

Compound	Exp	Calculated IEFPCM/MST	Calculated EC_RISM	Δp*K*_a_ EC_RISM	Calculated TZVP_QM	Δp*K*_a_ TZVP_QM
SM25	4.49	7.24	5.42	− 0.93	7.34	− 2.85
SM27	10.45	12.34	10.17	0.28	7.65	2.80
SM37	10.33	8.11	9.95	0.38	6.77	3.56
SM42	6.62	4.86	5.59	1.03	7.45	− 0.83

This analysis points out the need to perform an adequate sampling of the conformational states available for the different species involved in the deprotonation reaction [44, 45]. In particular, since our approach relied on the sampling performed for the neutral compounds (see above), the population of conformers obtained for ionized species may be inaccurate for some compounds, affecting the final estimate of the macroscopic p*K*_*a*_. Nevertheless, one must also keep in mind the intrinsic errors of the gas phase and solvation contributions to the aqueous free energy change for the deprotonation of the different microstates. At this point, the uncertainty of the IEFPCM/MST model in predicting the hydration free energy for simple neutral molecules amounts, on average, to 0.7 kcal/mol, but can be sensibly larger for charged compounds [46, 47]. This would then represent an additional difficulty for the proper estimation of the free energy change determined for microscopic deprotonation equilibria, challenging the ability of QM-based continuum solvation models to yield p*K*_*a*_ estimates with an uncertainty below 1 p*K*_*a*_ unit.

Overall, the results support the suitability of our QM-based approach for computing log *P* and p*K*_a_ properties. SAMPL6 blind challenge mainly relied on rigid compounds [38], but SAMPL7 presented more complex compounds considering both chemical diversity and flexibility [21]. In the blind challenges mentioned above, the Frog tool has been used to explore the conformational space in our QM workflow mainly due to the good balance between computational cost and accuracy of the conformer ensemble [25]. Ongoing research in our group is seeking to explore protocols for characterizing the conformer generation based on multilevel strategies [45], since the proper sampling of the conformational space is a crucial issue that can directly impact the reliable prediction of physicochemical properties [48–50]. The other two critical components of our QM approach are the calculation of the internal energy of the generated conformers and the inclusion of solvation effects, which are relevant in determining the accuracy of the relative stabilities of conformers in condensed phases. For example, extrapolation of the MP2 energies to complete basis set or the inclusion of higher-level electron correlation corrections, like coupled cluster with single and double substitutions (CCSD), could improve the accuracy of our protocol by several tenths of kcal/mol when computing deprotonation free energies or relative conformer stabilities [33, 51]. The improvement of solvation effects is more complicated, as there is no systematic strategy to improve the accuracy of the results given the empirically parametrized nature of continuum models. Nevertheless, the performance obtained in the SAMPL6 and SAMPL7 challenges shows close agreement with the results obtained in previous studies [16, 22, 32, 52] for rigid compounds, thus lending confidence to the computational protocol used in this study.

After checking and considering the different drawbacks of our workflow, we consider that further improvements should be focused on two computational aspects that may affect the prediction of physicochemical properties. The first deals with obtaining a proper sampling of the conformational space available for drug-like compounds in water and *n*-octanol (or by extension other organic solvents), as it is reasonable to expect that distinct conformational ensembles will be adopted depending on the chemical features present in flexible compounds. In this context the exhaustiveness in sampling the whole conformational space can be calibrated through the analysis of the conformations sampled with other techniques, such as Molecular Dynamics simulations. The second is related to the capability of continuum solvation models to provide an accurate description of specific (i.e., hydrogen bonding) and nonspecific (i.e., bulk solvent electrostatic screening) interactions with solvent molecules, which is challenging for charged molecules. In this sense, the usage of cluster-continuum solvation models may lead to meaningful improvement with respect to pure continuum solvation models for modeling diverse chemical process in solution [53].

## Conclusions

The results obtained in the SAMPL7 physical properties challenge has revealed the reliability of the IEFPCM/MST method to provide accurate estimates of both log *P* and p*K*_*a*_, which are relevant properties for understanding the pharmacokinetics of bioactive compounds. Nevertheless, the analysis of the results also points out that a major source of error comes from an improper weight of the conformational preferences of some compounds, particularly regarding the population distribution of ionized forms. In contrast, the prediction of the log *P* value resulted to have a marked deviation in one out of 22 compounds, though this marked deviation was also shared by a significant number of methods. Future modifications and improvements will be centered in finding an efficient approach for gaining better definition of the conformational space of flexible compounds in *n*-octanol and in water as well as to estimate the hydration free energies of charged species.

## Supplementary Information

Below is the link to the electronic supplementary material.

Supplementary file1 (DOCX 5939 kb)
