# Supplementary material for: Prediction of n-octanol/water partition coefficients and acidity constants (pKa) in the SAMPL7 blind challenge with the IEFPCM-MST model
Source: J Comput Aided Mol Des. 2021 Jul 10;35(7):803–11. doi: 10.1007/s10822-021-00394-6 (PMC8295120; doi:10.1007/s10822-021-00394-6)
Supplement: Supplementary file 1 — Supplementary file1 (DOCX 5939 kb) [file 10822_2021_394_MOESM1_ESM.docx]

**Supporting Information**

**Prediction of *n*-octanol/water partition coefficients and acidity constants (*pK_a_*) in the SAMPL7 blind challenge with the IEFPCM-MST model**

Antonio Viayna^1,*^, Silvana Pinheiro^2^, Carles Curutchet^3^, F. Javier Luque^1^, William J. Zamora^4,5*^

^1^ Department of Nutrition, Food Sciences and Gastronomy, Faculty of Pharmacy and Food Sciences, Institute of Biomedicine (IBUB), and Institute of Theoretical and Computational Chemistry (IQTC-UB), University of Barcelona (UB), Avda. Prat de la Riba, 171, 08921-Santa Coloma de Gramenet

^2^ Institute of Exact and Natural Sciences, Federal University of Pará, 66075-110 Belém, Pará, Brazil

^3^ Department of Pharmacy and Pharmaceutical Technology and Physical Chemistry, Faculty of Pharmacy and Food Sciences, and Institute of Theoretical and Computational Chemistry (IQTC-UB), University of Barcelona, Av. de Joan XXIII, 27-31, 08028-Barcelona

^4^ School of Chemistry & Faculty of Pharmacy, University of Costa Rica, San Pedro, San José, Costa Rica

^5^ Advanced Computing Lab (CNCA), National High Technology Center (CeNAT), Pavas, San José, Costa Rica

* Corresponding author: toniviayna@ub.edu

* Corresponding author: william.zamoraramirez@ucr.ac.cr

**Figure S1**. Root-mean square error (RMSE) of the 17 submissions to the *n*-octanol/water log *P* prediction challenge. Taken from [SAMPL7 repository](file:///Users/barna/Documents/18.SAMPL7/SAMPL7%20repository) (<https://github.com/samplchallenges/SAMPL7>).

**Figure S2**. Root-mean square error (RMSE) of the 6 submissions provided by physical methods to the *n*-octanol/water log *P* prediction challenge. Taken from [SAMPL7 repository](file:///Users/barna/Documents/18.SAMPL7/SAMPL7%20repository) (<https://github.com/samplchallenges/SAMPL7>).

**Figure S3**. Mean absolute error for the 22 compounds of the SAMPL7 dataset determined from the data reported for the total of 17 submissions. Taken from [SAMPL7 repository](file:///Users/barna/Documents/18.SAMPL7/SAMPL7%20repository) (<https://github.com/samplchallenges/SAMPL7>).


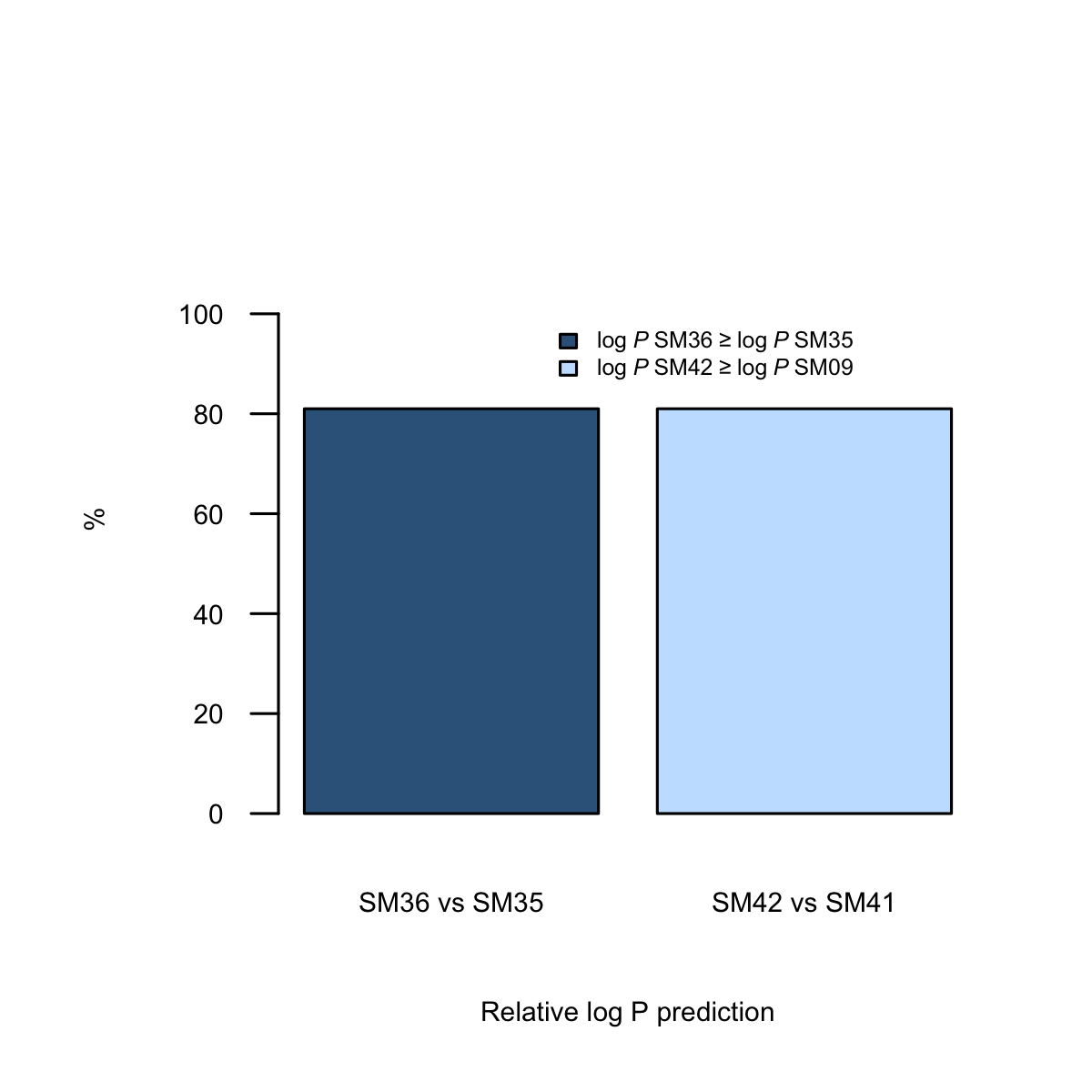


**Figure S4**. Relative log *P* prediction between the pairs SM36-SM35 and SM42-SM41 for all submissions in the challenge.

**
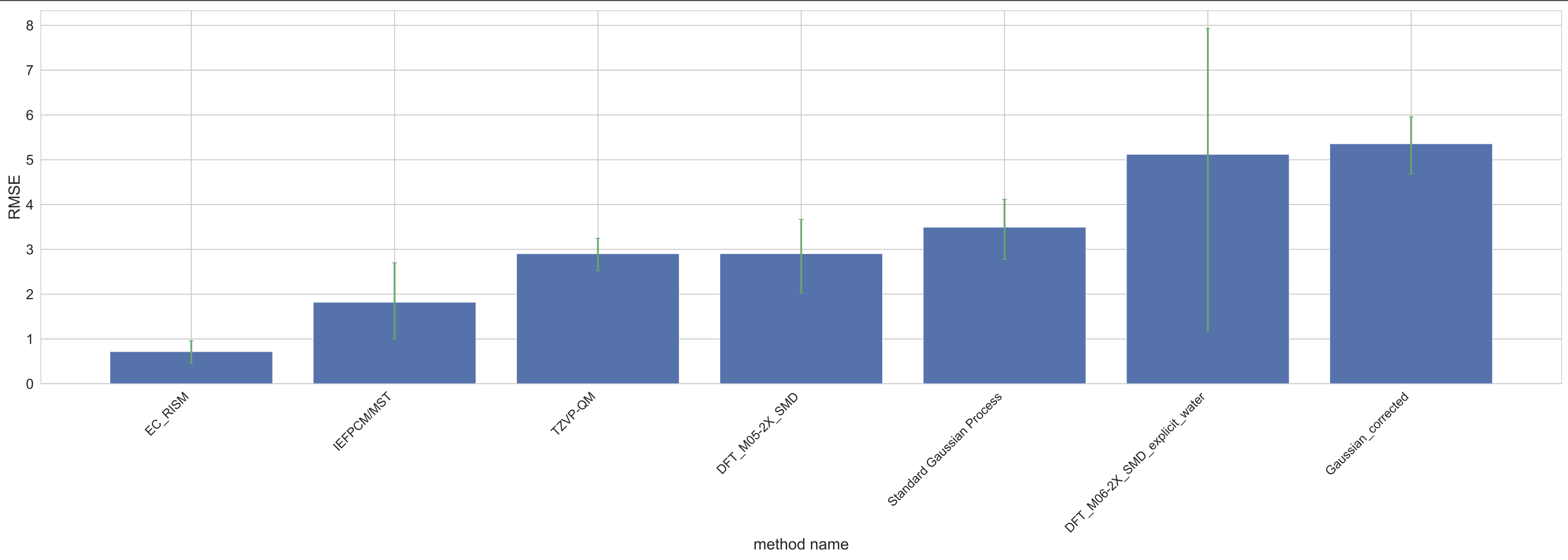
**

**Figure S5**. Root-mean square error (RMSE) of the 7 submissions to the p*K*_a_ challenge. Taken from [SAMPL7 repository](file:///Users/barna/Documents/18.SAMPL7/SAMPL7%20repository) (<https://github.com/samplchallenges/SAMPL7>).

**Table S1**. Comparison between the macroscopic p*K*_a_ values reported in the original submission^a^ and those determined by the organizers and reported in SAMPL7 website analysis.^b^

| Compound | p*K*_a_  (original submission) | p*K*_a_  (website) | Δp*K*_a_  (O.S. – W) |
| --- | --- | --- | --- |
| SM25 | 7.24 | 7.18 | 0.06 |
| SM26 | 4.52 | 4.48 | 0.04 |
| SM27 | 12.34 | 12.23 | 0.11 |
| SM28 | 16.12 | - | - |
| SM29 | 11.51 | 11.40 | 0.09 |
| SM30 | 11.00 | 10.90 | 0.10 |
| SM31 | 10.84 | 10.74 | 0.10 |
| SM32 | 11.95 | 11.84 | 0.11 |
| SM33 | 10.69 | - | - |
| SM34 | 10.64 | 10.79 | -0.15 |
| SM35 | 10.28 | 10.19 | 0.09 |
| SM36 | 9.20 | 9.12 | 0.08 |
| SM37 | 8.11 | 4.21 | 3.90 |
| SM38 | 9.82 | 9.73 | 0.09 |
| SM39 | 8.85 | 8.77 | 0.08 |
| SM40 | 8.26 | 8.18 | 0.08 |
| SM41 | 5.13 | 5.09 | 0.04 |
| SM42 | 4.86 | 4.82 | 0.04 |
| SM43 | 4.43 | 4.39 | 0.04 |
| SM44 | 7.09 | 7.02 | 0.07 |
| SM45 | 7.37 | 7.30 | 0.07 |
| SM46 | 5.56 | 5.51 | 0.05 |
| Mean excluding SM37 | - | - | 0.06 |
| Mean including SM37 | - | - | 0.25 |

^a^*https://github.com/samplchallenges/SAMPL7/blob/master/physical_property/pKa/analysis/relative_microstate_free_energy_predictions/pKa-IEFPCMMST-1.csv*)

^b^[*https://github.com/samplchallenges/SAMPL7/blob/master/physical_property/pKa/analysis/macrostate_analysis/analysis_outputs_ranked_submissions/pKa_submission_collection.csv*](https://github.com/samplchallenges/SAMPL7/blob/master/physical_property/pKa/analysis/macrostate_analysis/analysis_outputs_ranked_submissions/pKa_submission_collection.csv)
